# The Relief of Intracranial Pressure in General Paralysis of the Insane, Tabes Dorsalis and Other Diseases by Lumbar Puncture1State Hospital Bulletin, July, 1896.

**Published:** 1896-10

**Authors:** Warren L. Babcock

**Affiliations:** Second Assistant Physician, St. Lawrence State Hospital


### Selections.

### THE RELIEF OF INTRACRANIAL PRESSURE IN GENERAL PARALYSIS OF THE INSANE, TABES DORSALIS AND OTHER DISEASES BY LUMBAR PUNCTURE.[1]

[Abstract.]

BY WARREN L. BABCOCK, M. D.,
Second assistant physician, St. Lawrence State Hospital.

THE surgical treatment of general paralysis has heretofore been confined to two operations, trephining and laminectomy. The past few months has witnessed the introduction of a third method looking toward the alleviation of the only indication for surgical interference, *i. e.*, increased intracranial pressure.

Trephining, for the relief of brain pressure caused by fluid, was first suggested by T. Claye Shaw in 1889 and the first operation performed in July, of that year, by Harrison Cripps. The procedure found an earnest advocate in J. Batty Tuke, who latterly suggested laminectomy with permanent drainage in its stead.

The first attempt to treat paresis surgically was by Charles G. Wagner, at Utica. He trephined a negro paretic in March, 1890, and, though the case died six months after operating, much temporary benefit was manifest by an amelioration of the prominent mental symptoms.

Surgical treatment, by any method whatever, looks toward the relief of pressure symptoms. These are caused by the great amount of fluid exuded into the lymph channels of the brain and

1. State Hospital Bulletin, July, 1896.

meninges as a result of the cortical and meningeal inflammation in the first stage of the process, supplemented later by the compensatory exudation which follows atrophy and sclerosis. The nature of the fluid is a mooted question. Macpherson and Wallace assume that it is at first an inflammatory exudate; that it occurs early and is primary to the sclerosis of nerve elements which follow. Bevan Lewis, who holds that the arterial changes are first to occur, claims that the excess of fluid is compensatory throughout the disease and follows the cortical nerve cell atrophy.

A resumé of the operative work, having as its object the removal of this fluid in excess, demonstrates in most cases some temporary improvement in the mental symptoms, such as headache, irritability, stupor, confusion of ideas, and in some instances has afforded relief from persistent hallucinations and troublesome delusions. Tuke reports two recoveries in the first stage in cases which have since had the diagnosis called into question. In a report of recent cases trephined at Sterling, Macpherson states that the course of the disease is evidently checked for many months by the surgical relief of pressure, and suggests "that if we could only manage to diagnosticate general paralysis at a sufficiently early stage, we might then by operative interference check its aggressive march."

The transient improvement afforded by operation apparently depends on (1) relief of pressure by removal of fluid; (2) greater opportunity for brain expansion and pulsation by removal of bone; (3) subsidence of meningeal inflammation by local depletion of blood-vessels, and (4) shock to nervous system as a direct result of operation.

*Locomotor Ataxia.*—How does the relief of this pressure alleviate ataxia? The following modes are at once suggested:

1. By simple mechanical relief of pressure to which the coördinating centers are subjected.

2. By improvement in the circulation of the motor centers of the cord, medulla and cortex.

3. The withdrawal of fluid and the consequent improvement in the circulation of the cortical arterioles, reëstablish, temporarily, the inhibitory functions of the higher centers.

The ataxia of the following case of tabes dorsalis was greatly benefited by puncture:

No. 2275.—Admitted October 3, 1895. Male; aged 68 years. Subacute mania with locomotor ataxia. Duration unknown. Ataxia well marked on admission and slowly progressed to almost complete paraplegia. When punctured, patient had been in bed for four months. Operation May 26, 1896 ; 95 c.c. clear cerebro-spinal fluid removed in sixty minutes. During operation he became irritable and disturbed, perspired freely, complained of headache, and respiration and pulse increased in frequency. Following day was confused, delusional and irritable. One week later patellar reflexes were apparent and he began to use his legs with the assistance of a chair. In a few days he walked without support to the dining-room and has continued to get about with but little difficulty since. Facial expression, formerly dull and blank, has become fairly intelligent in its expression. General physical health has improved ; he has gained in weight and grown considerably stronger. When the advancement toward a moderate degree of health in a case as far advanced in tabes as the above is so apparent, it is evident that the method is worthy of a more extended trial.

*Diagnostic and Therapeutic Possibilities of Lumbar Puncture.* —Experimental puncture is a justifiable procedure in doubtful cases of meningitis, even though symptoms of pressure are absent. Precipitation of sediment in fluid and staining may demonstrate the presence of germs in the exudate. Albert Frankel reports a case in which the diagnosis of tubercular meningitis was made from an examination of the fluid thus obtained. Furbinger reports similar cases. Stadelman found the diplococcus of croupous pneumonia in the exudate of one case. More recently, Caille, in discussing tubercular meningitis, suggests laying bare the dura by trephine and irrigating the sub-arachnoid space from the seat of puncture upward, the fluid escaping through opening in dura. This would also be applicable to any meningeal inflammation, including infectious cerebro-spinal meningitis and the acute meningitis of acute delirium. The utility of this form of spinal lavage in general paralysis is doubtful. No indications suggest its possible efficacy in this disease. The writer, however, has the subject under investigation and hopes to find material for a further report.

The clinical symptoms of paresis that indicate the operation are those apparently caused by the increased intracranial pressure. The principal symptomatic indication is the frequently recurring periods of the second and third stages of deep stupor, which is almost entirely due to a sudden or compensatory increase in the amount of cerebro-spinal fluid, and partially masks or veils the underlying dementia. Upon its removal the true condition of the patient is often made apparent. Headache, vomiting, vertigo, convulsion, suffused face and high arterial tension are symptoms that suggest increased cerebral tension, and in the experience at least of the writer was relieved, temporarily at least, by puncture.

The author then narrates the histories of 12 cases of general paralysis, 1 case of locomotor ataxia, 1 of stuporous melancholia, 2 of simple melancholia with pressure symptoms, 1 of organic dementia, 1 of status epilepticus and 1 of acute delirium which were certainly relieved by this operation.

*Character of the Fluid Obtained by Puncture.*—The specific gravity of the cerebro-spinal fluid in general paralysis is generally lower than normal. In one case in the third stage it was found to be 1003. From this low figure it ranged up to 1010, one case only reaching that figure, which is considered to be the normal. The average for the twelve cases was less than 1007. The specific gravity of the fluid from cases other than paresis closely approximated the normal as will be seen by a reference to the table.

The reaction of the fluid was neutral in over 90 per cent. of the paretics and in three-fourths of the remaining cases. The normal reaction of the fluid is neutral or faintly alkaline. In no case did it show great alkalinity or even faint acidity. Bevan Lewis states that he has found it to be acid in reaction in paretics dying in third stage. He is evidently in error and the acidity he noticed was probably a post mortem change.

A careful study of results brings out the following conclusions:

1. Lumbar puncture affords temporary relief from pressure symptoms in over 50 per cent. of cases of paresis submitted to the operation.

2. The most beneficial effects are manifest over motor incoördination, *i. e.*, ataxia, tremors, etc.

3. Analysis of the fluid obtained in paresis shows that it contains an inflammatory product (albumin) throughout all stages.

4. It may be of benefit in locomotor ataxia, status epilepticus or organic cerebral disease and deserves further trial in these cases.

5. It presents excellent diagnostic possibilities, particularly in meningeal inflammations.

6. It does not sufficiently benefit melancholia with pressure symptoms to warrant its use in this disease, and lastly

7. Re-accumulation usually occurs within from three to ten weeks, when a second or even a third puncture is indicated if patient's condition admits.

